# V_o2peak_, Ve/V_CO2_, and Cardiac Remodeling Correlate with Long-Term Cardiovascular Outcome in Heart Failure Patients

**DOI:** 10.3390/jcdd12050174

**Published:** 2025-05-02

**Authors:** Antonio Pagliaro, Luna Cavigli, Roberta Molle, Elisabetta Iardino, Francesca Anselmi, Francesca Righini, Luca Martini, Valerio Zacà, Giulia Elena Mandoli, Maria Concetta Pastore, Marta Focardi, Matteo Cameli, Sonia Bernazzali, Massimo Maccherini, Marco Chiostri, Flavio D’Ascenzi, Serafina Valente

**Affiliations:** 1 Division of Cardiology, Department of Medical Biotechnologies, University of Siena, 53100 Siena, Italy; 2 Department of Cardiac Surgery, University of Siena, 53100 Siena, Italy; 3 SOD Fisiopatologia Respiratoria, Dipartimento delle Specialistiche Mediche, Az. USL Toscana Centro, 50137 Firenze, Italy

**Keywords:** heart failure, left ventricular ejection fraction, cardiopulmonary exercise testing, heart transplantation, prognostic stratification, cardiac remodeling

## Abstract

Accurate prognostic stratification in patients with chronic heart failure and reduced ejection fraction (HFrEF) remains a significant clinical challenge. Many different parameters, including left ventricular (LV) and right ventricular (RV) function and cardiopulmonary exercise testing (CPET) parameters, are available in the literature. LV ejection fraction (LVEF) is the most used parameter in clinical practice. This study aimed to analyze CPET and echocardiographic data in patients under evaluation for heart transplantation (HTx) to identify the parameter that best correlates with cardiac events. Methods and Results. Echocardiography and CPET were performed in patients with HFrEF under evaluation for HTx. The population comprised 170 patients (mean age: 55 ± 9 years; 88% male; non-ischemic etiology: 63%). LVEF was 30.4 ± 7.6%, peak oxygen uptake (Vo_2peak_) was 17.08 ± 4.6 mL/Kg/min; minute ventilation (VE)/carbon dioxide production (Vco_2_) slope was 34.8 ± 8.7. During a follow-up of 4 ± 1 years, 37 hospitalizations, 4 deaths, 14 HTx, and 5 LV assist device implantation occurred. Patients who experienced major events had a lower Vo_2peak_ (*p* < 0.005), higher VE/Vco_2_ slope (*p* < 0.005), greater LV end-systolic diameter (*p* < 0.005), and RV end-diastolic diameter (*p* < 0.005) than patients without events. Conversely, LVEF did not differ between these two groups. VE/Vco_2_ slope and RV dimensions significantly correlated with hard cardiac events (*p* = 0.019 and *p* = 0.008, respectively). Conclusions. In patients with HFrEF, parameters quantifying the system reserve (i.e., Vo_2peak_ and VE/Vco_2_ slope) and those demonstrating advanced biventricular remodeling may help stratify the risk of cardiac events. Conversely, LVEF showed a limited prognostic value in this setting.

## 1. Introduction

Heart failure (HF) is a challenge for cardiologists and modern society. Its prevalence and management costs underscore the importance of appropriate management strategies for patients with HF [1]. Echocardiography plays a fundamental role in the diagnosis and provides a preliminary stratification of HF in relation to left ventricular (LV) ejection fraction (LVEF), as suggested by current guidelines [1]. However, as an indicator of cardiac function, EF has several limitations. It is calculated as the ratio of stroke volume to end-diastolic volume, with its estimation typically relying on geometric assumptions derived from linear or two-dimensional measurements. Moreover, LVEF is influenced by both preload and afterload, can vary significantly depending on loading conditions, has moderate reproducibility, and represents only one of many risk factors in HF patients [2]. Therefore, since the risk estimation of future events is necessary for these patients to optimize the clinical management and use of medical therapy and/or devices, LVEF seems unsatisfactory for these purposes [3,4,5]. To overcome these limitations, physicians should refer to a multiparametric evaluation that considers personal and clinical history, echocardiography, and laboratory and functional data. Among functional tests, cardiopulmonary exercise testing (CPET) has demonstrated a relevant role in HF patients, being a cornerstone also in the selection of patients who are candidates for heart transplantation (HTx) and for exercise prescription and monitoring of training programs [6,7,8,9,10]. Despite the established role of CPET and echocardiography in the evaluation of HF patients, further investigation is needed to understand better how their combined interpretation within a multiparametric approach may improve risk stratification, guide clinical decisions, and optimize personalized care. This study aimed to evaluate the correlation and the prognostic relationship between CPET and echocardiographic data with the occurrence of major cardiovascular cardiac events (MACE) during the follow-up in patients affected by HFrEF.

## 2. Methods

All patients enrolled in this study were referred to the Cardiac Department of the University Hospital Santa Maria alle Scotte in Siena from 1 January 2012 to 1 October 2016. CPET data were integrated with the results obtained by echocardiography, performed within 7 days of the CPET examination. Patients included in this study had to fulfill the following inclusion criteria: a previous diagnosis of chronic HFrEF (LVEF ≤ 40%), clinically stable, and on top of optimal medical and device-related treatment. Exclusion criteria were: LVEF > 40%, ongoing up-titration of medical therapy, achievement of a peak respiratory exchange ratio (RER) < 1.00 (to ensure that only tests reflecting adequate effort were included in the analysis), presence of a pattern of ventilatory limitation (preserved first ventilatory threshold, desaturation during effort, ventilatory reserve exhaustion), ventilatory reserve erosion. Regarding ventilatory limitation, patients showing clear ventilatory patterns suggestive of primary pulmonary limitation (e.g., abrupt ventilation drop or poor tidal volume increase) were excluded. For ventilatory reserve, we calculated it as the ratio between the maximal minute ventilation (VE) achieved during exercise and the estimated maximal voluntary ventilation (MVV). A ventilatory reserve of less than 15% was considered indicative of significant ventilatory limitation, and such cases were excluded to avoid confounding pulmonary factors.

Patients were followed for 4 ± 1 years. Information about the occurrence of MACE was collected during this period. MACEs included hospitalization for HF, cardiovascular death, HTx, and LV assist device (LVAD) implantation. Information on MACE was obtained by analyzing available medical records, follow-up clinical visits, and phone interviews.

After the rationale and protocol of this study were explained, the participants gave their written informed consent. The project was approved by the local Ethical Committee of the University of Siena.

### 2.1. CPET Protocol

All patients underwent symptom-limited CPET and were carefully instructed to achieve maximal effort, and all of them were familiar with the 10-point Borg fatigue scale [6,10]. A standard 12-lead ECG was recorded at rest, and the ECG was continuously monitored during the test. Blood pressure was measured using a manual sphygmomanometer every 2 min. All patients were limited by fatigue. These CPET data were acquired on a cycle ergometer (Quark CPET, CosMed USA Inc., Concord, CA, USA) equipped with OMNIA 1.6.5 software (CosMed USA Inc., Concord, CA, USA). At the beginning of each test day, a gas and volume calibration was performed according to the manufacturer’s instructions [11]. The exercise test (ramp protocol) included a 1-min pre-exercise resting period sitting upright on the bike and a 2-min unloaded warm-up cycling phase, useful to allow their adaptation to the mask and overcome a possible psychogenic hyperventilation phase, aiming to reach a starting RER of 0.70–0.85. We chose a ramp protocol because it allows for a smoother, individualized increase in workload, improving the precision of physiological measurements compared with step protocols; it also minimizes abrupt changes that could lead to early fatigue in HFrEF patients. The warm-up phase was followed by an incremental exercise cycling period whose workload increase was chosen according to the patient’s clinical status (5–15 W per minute) and aiming to complete the CPET within 8–12 min, as recommended [6,10,11]. While we aimed for an 8–12 min test duration, a few cases (<5%) fell outside this range because of exercise intolerance. To ensure that peak of oxygen uptake (Vo_2peak_) was attained, at least two of the following criteria had to be met: (1) maximal heart rate (HR) at a value close to 90% of the theoretical maximal HR, (2) RER ≥ 1.10, and (3) pedal rate note maintained at least at 60 rpm at each level of exercise.

Minute ventilation (VE), Vo_2_, carbon dioxide production (Vco_2_) values, and all the other variables of the test were acquired through a turbine transducer system (with breath-by-breath mode) and averaged every 10–15 s. Vo_2peak_ was intended as the average among the highest values of oxygen consumption measured in the last 30 s of the peak of the exercise and was expressed as VO_2peak_ indexed for body weight (mL/kg/min) and as a percent of predicted Vo_2peak_. The predicted Vo_2_ values were calculated using Hansen–Wasserman equations (corrected for age, sex, weight, and height) [12]. The first ventilatory threshold (VT_1_) was determined according to three validated methods to determine VT_1_ from incremental exercise test data: (1) modified V-slope method; (2) ventilatory equivalent method (VE/Vo_2_ method); (3) end-tidal O_2_ pressure method (P_et_o_2_). The VE versus W relationship was also considered [13,14]. The V-slope was the reference method for VT_1_ determination, and this point was checked with the point obtained in the other graphs [14].

The Vo_2_/work slope and the oxygen uptake efficiency slope (OUES) were automatically calculated by Quark COSMED CPET equipment software 1.6.5. VE/Vco_2_ slope was obtained through an automatic analysis performed by the software, using acquired values of Vo_2_ and Vco_2_ and excluding initial sampling from both pre-exercise hyperventilation phases and recovery phases, measuring it throughout the entire exercise duration. Peak systolic arterial pressure (PSAP) was considered the maximum pressure value during the exercise phase before recovery. Heart-rate recovery (HRR) was intended to be the difference between the maximal HR minus HR after a 1-min recovery.

### 2.2. Echocardiography

Expert cardiologists performed an echocardiographic examination using high-quality echocardiography (Philips IE33 Ultrasound Machine, Amsterdam, the Netherlands) equipped with an M4S 1.5-MHz to a 4.0-MHz transducer, and a one-lead ECG was continuously displayed. The echocardiographic examinations were performed by expert cardiologists with over 10 years of experience in cardiovascular imaging. LV end-diastolic (EDD) and end-systolic (ESD) diameters were assessed, LV volume measurements were calculated from the apical four- and two-chamber views using the modified Simpson’s rule and LVEF was calculated according to the current guidelines for chamber quantification [15]. Right ventricular mid-cavity end-diastolic diameter (EDD) and RV function were assessed as recommended by current guidelines [15]. The pulmonary arterial systolic pressure (PAPs) was estimated by sampling the trans-tricuspid regurgitation peak velocity through continuous wave Doppler imaging and using modified Bernoulli’s equation [15].

### 2.3. Statistical Analysis

The normal distribution of all continuous variables was examined using the Shapiro–Wilk test, and data were presented as mean ± SD. Categorical variables are expressed as percentages. According to the data distribution, the unpaired *t*-test and the Mann–Whitney test were used to assess the significance between groups. The chi-squared test was used for nominal data. The correlation between VE/V_CO2_ and echocardiographic measurements was performed using Pearson’s or Spearman’s correlation coefficient as appropriate for the data distribution. A *p*-value < 0.05 was considered statistically significant. Statistics were performed using SPSS, version 21.0 (Statistical Package for the Social Sciences Inc., Chicago, IL, USA).

## 3. Results

### 3.1. Demographic and Clinical Data

A total of 170 patients fulfilled the established inclusion/exclusion criteria. The demographic and clinical characteristics of this study population are reported in Table 1. Most patients were males (88%), and the etiology of HF was non-ischemic (idiopathic/post-myocarditis) in 63% of patients, while 37% had post-ischemic etiology. The drug therapy included beta-blockers in 91% of patients with a prevalence of bisoprolol and carvedilol (64% and 26%, respectively). Angiotensin-converting enzyme inhibitors/angiotensin receptor blockers (ACE-I/ARB) were used in 87% of patients, mineralocorticoid receptor antagonist (MRA) in 82%, ivabradine in 15% and loop diuretics in 84%. Forty percent of patients had cardiac resynchronization and defibrillator implantation (CRT-D), while 38% were implantable cardioverter defibrillator (ICD) carriers.

Data obtained by CPET are reported in Table 2. Neither hypertensive response during exercise nor significant O_2_ desaturation was observed. The patients had a reduced functional capacity with a mean Vo_2peak_ value of 17.1 ± 4.6 mL/kg/min. The Vo_2_/work slope was 9.5 ± 1.4 mL/min/W, while the OUES was 1.7 ± 0.6 L/min.

This study’s population had a reduced LVEF (mean EF 30.4 ± 7.6%) with a prevalence of dilatation remodeling (LV EDD: 67 ± 9 mm, LV ESD: 54 ± 12 mm). The mean RV EDD was 35 ± 7 mm, with an sPAP = 34 ± 12 mmHg. The mean tricuspid annular post-systolic excursion was TAPSE: 19 ± 4 mm.

### 3.2. Follow-Up and Cardiovascular Events

**Figure 1:**
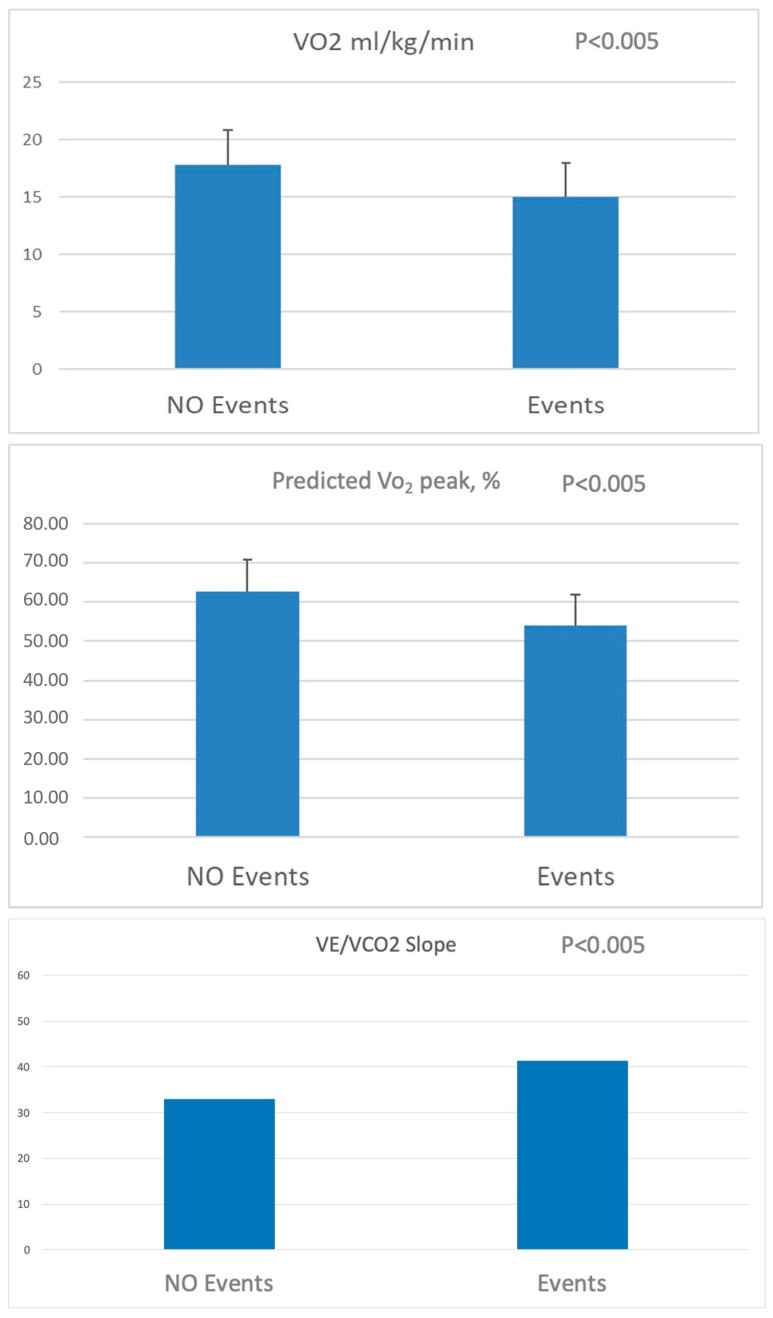
Comparison of main cardiopulmonary exercise testing data between patients with heart failure with and without cardiovascular events during the follow-up.

**Figure 2:**
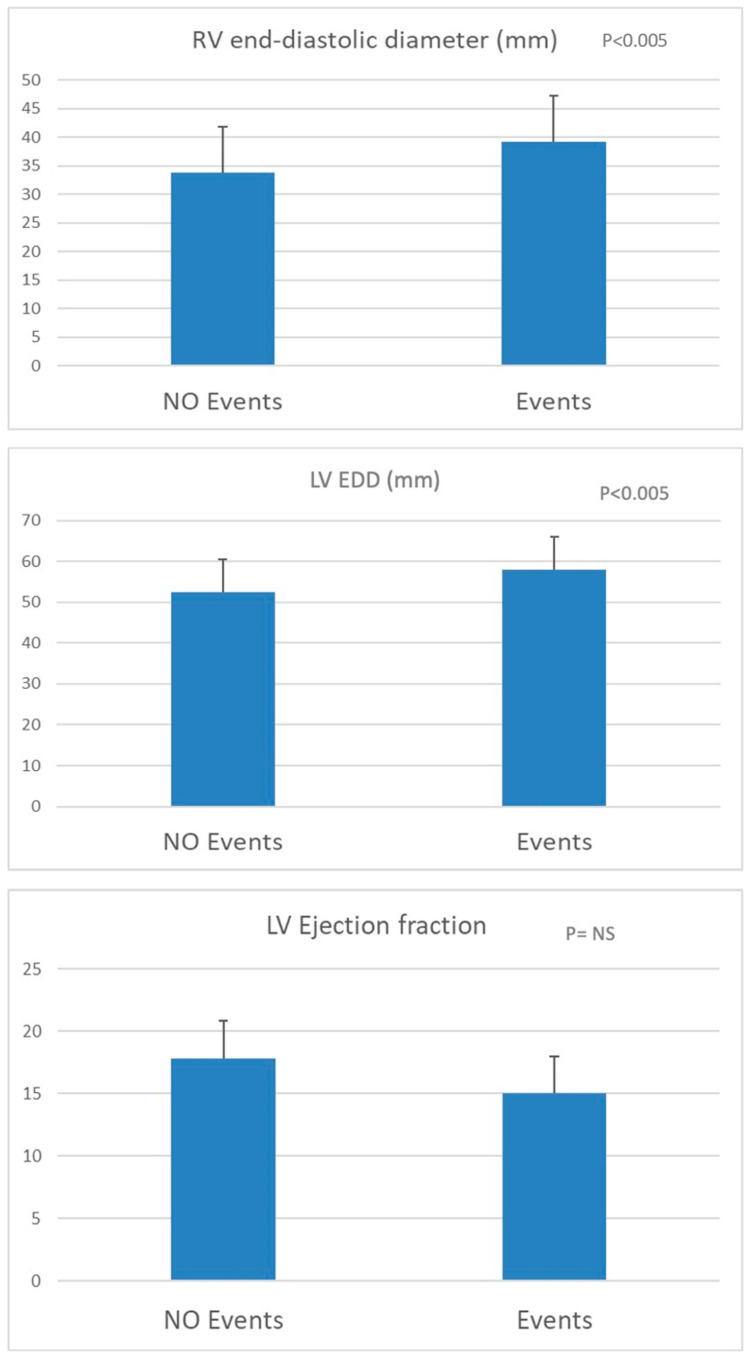
Comparison of main echocardiography data between patients with heart failure with and without cardiovascular events during the follow-up.

Prognostic information was obtained from 133 patients. During the observation period, 60 adverse events were observed: 37 hospitalizations for HF, 14 HTx, 5 LVAD implants, and 4 deaths due to cardiac causes. One additional death occurred because of a non-cardiac cause but was excluded from the final analysis. This study’s population was divided into two groups: patients who remained free of events and those who experienced events during follow-up. Regarding these CPET data, both the VO_2_ peak indexed to body weight and expressed as a percentage of the predicted value, as well as the VE/V_CO2_ slope, showed significant differences between the two groups (Figure 1). Similarly, significant differences were observed in LV ESD and mean RV EDD. Notably, no significant difference was found in LVEF between the groups (Figure 2).

Patients experiencing cardiovascular events during the follow-up were those with a mean value of Vo_2peak_ < 15 mL/kg/min, % predicted Vo_2_ < 55%, VE/Vco_2_ slope > 41, LV ESD > 52 mm, and RV EDD > 39 mm. In the multivariate analysis, RV EDD significantly correlated with hospitalization (*p* = 0.04). A correlation between HTx and %predicted Vo_2_ was observed (*p* = 0.027). Considering all the adverse events (hospitalization, HTx, and LVAD implantation), a significant correlation was found for RV EDD and VE/Vco_2_ slope (*p* = 0.008 and *p* = 0.019, respectively).

The main results of this study are shown in the Central Illustration.

## 4. Discussion

This study analyzed CPET and echocardiographic data in patients under evaluation for HTx to determine the parameters that best correlate with cardiac events. We demonstrated that in patients with HFrEF, parameters quantifying the system reserve (i.e., Vo_2peak_ and VE/Vco_2_ slope) and those demonstrating advanced biventricular remodeling may help stratify the risk of cardiac events in this population. Conversely, LVEF showed a limited prognostic value in this setting.

Consistent with previous studies, we found that V_O2peak_ is a powerful predictor of adverse outcomes in HFrEF patients, including those on beta-blocker therapy, and it remains robust even when adjusted for natriuretic peptides and other clinical variables [7,16,17]. Indeed, CPET is useful for defining the optimal timing of HTx in patients with severe LV dysfunction [16,18], and it generally provides a prognostic and diagnostic stratification for patients with HFrEF [7]. The deterioration of functional capacity, which corresponds to a Vo_2peak_ < 14 mL/kg/min or < 12 mL/kg/min for patients on beta-blocker therapy or percent of predicted (<50%) V_O2peak_ in young patients (<50 years) and women, is used for the indication to HTx [7,18]. As previously shown, patients below these thresholds have significantly lower 1-year survival rates compared with those who undergo HTx, underscoring the clinical utility of this parameter in guiding advanced HF therapies [16]. Conversely, individuals with a V_O2peak_ exceeding the threshold demonstrated a lower one-year mortality rate. Notably, these groups had no discernible variance in resting LVEF or cardiac index. The severe impairment of functional capacity shown by 6-MWT distance < 300 m or V_O2peak_ < 14 mL/kg/min contributes to the early recognition of patients with advanced or stage D HF who have become refractory to traditional therapies and are suitable for advanced treatments, which could influence survival [19].

In agreement with prior reports, our study supports the prognostic relevance of the VE/V_CO2_ slope in chronic HF, particularly in predicting major cardiac events such as death, LVAD implantation, or HTx [7,20]. However, while prior studies have often emphasized V_O2peak_ in isolation, our study provides additional evidence that combining V_O2peak_ with VE/V_CO2_ slope > 41 further improves risk discrimination. This threshold is slightly higher than the commonly reported cutoffs (>34–36) [21].

A correlation between RV function and functional capacity to predict prognosis among patients with advanced HF has already been established [22,23]. More recent studies have shifted focus toward the interaction between the RV and pulmonary circulation. During exercise, in both HFrEF and HFpEF patients, impaired RV performance is interpreted as an inability to increase cardiac output, often reflected by a low Vo_2peak_ [24,25]. This limitation is primarily due to increased RV afterload associated with pulmonary arterial hypertension, which closely correlates with RV systolic function during exertion. Moreover, the VE/Vco_2_ slope reflects the relationship between RV and pulmonary circulation; ventilator inefficiency due to the rise of ventilation equivalent for the increase in carbon dioxide production is a consequence of a decrease in the ventilation/perfusion (*V*/*Q*) ratio. The reduction in the *V*/*Q* ratio is probably related to RV failure to accommodate increased blood flow and allow pulmonary capillary recruitment during exercise [26,27,28]. Despite the same RV function at rest, the VE/Vco_2_ slope value shows RV’s ability to cope with the hemodynamic changes during exercise (a low VE/Vco_2_ slope value is related to better RV performance). Hence, the VE/Vco_2_ slope adds information to RV static evaluation and predicts its contractile reserve [29]. More recent data confirm this correlation between right ventriculo-arterial coupling and CPET variables in patients with HFpEF and pulmonary hypertension [30,31].

Our study also demonstrates that echocardiographic parameters of biventricular dimensions describing myocardial remodeling (i.e., LV ESD and RV EDD) are useful for prognostic stratification, while LVEF does not add relevant information and cannot stratify the risk in this setting. Conversely, in these patients, variables describing extreme LV remodeling (i.e., LV ESD > 52 mm) and RV remodeling (i.e., RV EDD >39 mm) or dynamic CPET parameters reflecting an estimation of cardiac reserve (V_O2peak_ and VE/Vco_2_ slope) provide relevant information for the risk stratification and predicting the prognosis of patients with HFrEF.

These findings align with previous research indicating that RV dilation is a marker of poor prognosis, associated with increased filling pressures, arrhythmic risk, and impaired LV function [32,33]. Moreover, RV size has been shown to have prognostic value in HFrEF patients, to contribute to an adverse outcome independently of RV dysfunction, and to predict mortality [32,33]. Similarly, LV enlargement is a powerful predictor of adverse outcomes such as all-cause death, CV death, HF hospitalization, and myocardial infarction in patients with HFrEF [34]. The most widely accepted explanation of LV enlargement as a predictor of CV outcomes is that LV enlargement is a compensatory mechanism for LV systolic dysfunction [34]. In a study by Ito et al., the investigators examined whether the risk linked to LV enlargement was independent of LVEF. They found that increased LV dimensions were significantly associated with higher all-cause mortality in patients with both a preserved (LVEF ≥ 25%) and severely reduced (LVEF < 25%) ejection fraction [34]. These findings suggest that the prognostic value of LV size is not merely a reflection of concomitant variations in LVEF. Rather, they highlight the need to consider LV enlargement as an independent risk factor, irrespective of EF [34]. Indeed, some studies demonstrated that patients with HFpEF had complication rates that were similar to those of patients with a reduced EF, including similar rates of cardiac arrest, acute coronary syndrome, renal failure, and admission to the intensive care or coronary care unit [3]. Conversely, other studies demonstrated that a decrease in EF below 45% is associated with an approximately 40% increase in all-cause mortality for every 10% reduction [35]. A notable divergence from previous research lies in our echocardiographic findings. While many studies continue to use LVEF as a primary stratification tool, we found that LVEF had limited prognostic value in this high-risk population. Indeed, the echocardiographic assessment of LVEF faces several limitations, including poor image quality, anatomical variability, and the need for manual or semi-automated tracing, all contributing to measurement inconsistency [2]. Since LVEF is derived from volume estimates, it is influenced by loading conditions and geometric assumptions, leading to variability and potentially inaccurate reflection of true contractility. For example, athletes may show slightly reduced LVEF because of larger ventricular volumes, while patients with hypertrophy may display unusually high values. In cases of ventricular dyssynchrony, such as left bundle branch block, the largest LV volume may not coincide with end-diastole, compromising the accuracy of LVEF measurement [2]. In conclusion, our findings largely confirm previous evidence on the utility of CPET variables in predicting outcomes in HFrEF while also highlighting the superior prognostic value of advanced biventricular remodeling over LVEF. Therefore, in the prognostic and therapeutic stratification of patients with HF, it is essential to carry out a multiparametric evaluation that does not only consider the EF.

### Limitations

The current analysis is retrospective, and data were collected from patients evaluated in a single center for advanced HF therapy, which may limit the generalizability of our findings to broader populations. Additionally, the retrospective nature of data collection introduces the potential for selection bias, as only patients who met specific inclusion criteria were included, potentially excluding those with more complex or atypical presentations. Moreover, we analyzed data about out-of-hospital or hospitalized patients. This may explain the young age, the prevalence of non-ischemic cardiomyopathy, and the moderately reduced functional capacity. However, the relatively small difference between our data and the CPET-derived prognostic cutoffs supports the quality of these present data and is in line with the current evidence.

Some echocardiographic-derived patient variables recently affirmed for RV evaluation (e.g., RV longitudinal strain) were unavailable. In particular, there is evidence about the role of RV longitudinal strain in evaluating RV in LVAD candidates and HFrEF patients [36]. However, in our study, the indirect evaluation of RV contractile reserve achieved with VE/V_CO2_ slope could overcome, at least partially, the limits derived from standard echocardiographic RV evaluation.

## 5. Conclusions

Optimal prognostic stratification in ambulatory patients with severe LV dysfunction remains a complex challenge. LVEF is important for many therapeutic goals, but it is insufficient alone. Our study demonstrates that combining variables that reflect advanced stages of heart disease—such as extreme LV remodeling with RV involvement—and those that assess the cardiopulmonary reserve, such as V_O2peak_ and VE/V_CO2_ slope, provides important additional prognostic information. These parameters offer valuable insights into the underlying pathophysiology, especially for patients with HFrEF, and can guide therapeutic decisions more accurately than LVEF alone. The clinical implications of our findings underscore the need for a multiparametric approach to patient evaluation, particularly in the context of HF management and heart transplantation decisions. By integrating echocardiographic measures with functional testing, such as CPET, clinicians can achieve a more comprehensive understanding of a patient’s condition, improving the ability to predict adverse events and tailor treatment strategies accordingly.

Future studies with larger, multi-center cohorts and prospective designs are needed to validate and expand upon our findings.

## Figures and Tables

**Table 1:** Clinical characteristics of this study’s population.

Total Patients, n	170
Age (years)	55 ± 10
Sex, Male, n (%)	149 (88)
Weight (kg)	81.4 ± 13.4
Height (cm)	173 ± 8
Ischemic DCM n (%)	63 (37)
NIDCM, n (%)	107 (63)
ICD, n (%)	65 (38)
CRT-D, n (%)	68 (40)
CRT-P, n (%)	1 (1)
Βetablockers, n (%)	154 (91)
ACE-inhibitors/ARBs, n (%)	148 (87)
ARNI, n (%)	3 (2)
MRI, n (%)	139 (82)
Diuretics, n (%)	143 (84)
Diuretic dose (mg)	75.9 ± 60.0
Ivabradine, n (%)	26 (15)

DCM: dilated cardiomyopathy; NIDCM: Non-ischemic dilated cardiomyopathy; ICD: implantable cardiac defibrillator; CRT-D: cardiac resynchronization therapy-defibrillator; CRT-P: cardiac resynchronization therapy-pacing; ACE-inhibitors: angiotensin-converting enzyme inhibitors; ARBs: angiotensin receptor blockers; ARNI: angiotensin receptor neprilysin inhibitors; MRI: mineral corticoid receptor inhibitors.

**Table 2:** Cardiopulmonary exercise tests and echocardiographic data collected in this study’s population.

V_O2_ mL/min	1367.7 ± 422.0
V_O2_ mL/kg/min	17.1 ± 4.6
%ppV_O2_	61.1 ± 14.0
VE/V_CO2_ slope	34.8 ± 8.7
RER	1.08 ± 0.09
V_O2_/Work mL/min/watt	9.5 ± 1.4
OUES L/min	1.7 ± 0.6
pSBP mmHg	132 ± 24
HRR, bpm	13 ± 7
LV EDD, mm	66 ± 9
LV ESD, mm	53 ± 11
LVEF, %	30 ± 7
RV EDD, mm	35 ± 7
TAPSE, mm	18 ± 4
PAPs, mmHg	34 ± 11

V_O2_: peak oxygen uptake; %ppV_O2_: percent predicted peak V_O2_; VE/V_CO2_ slope: minute ventilation/carbon dioxide production slope; RER: respiratory exchange ratio; OUES: oxygen uptake efficiency slope; pSBP: Peak systolic blood pressure; HRR: heart rate recovery. LV: Left ventricular; EDD: end-diastolic diameter; ESD: end-systolic diameter; EF: ejection fraction; RV: Right ventricular; PAPs: pulmonary arterial systolic pressure.

## Data Availability

Data are available upon reasonable request.
